# ERK-mediated NF-κB activation through ASIC1 in response to acidosis

**DOI:** 10.1038/oncsis.2016.81

**Published:** 2016-12-12

**Authors:** B Chen, J Liu, T-T Ho, X Ding, Y-Y Mo

**Affiliations:** 1 Department of Urology, Affiliated Hospital of Jiangsu University, Zhenjiang, China; 2 Cancer Institute, University of Mississippi Medical Center, Jackson, MS, USA; 3 Department of Emergency Medicine, the First Affiliated Hospital of Guangxi Medical University, Nanning, China; 4 Department of Radiation Oncology, University of Mississippi Medical Center, Jackson, MS, USA; 5 College of Life Sciences, Zhejiang Sci-Tech University, Hangzhou, China; 6 Department of Pharmacology/Toxicology, University of Mississippi Medical Center, Jackson, MS, USA

## Abstract

Acidic microenvironment is a common feature of solid tumors. We have previously shown that neuron specific acid-sensing ion channel 1 (ASIC1) is expressed in breast cancer, and it is responsible for acidosis-induced cellular signaling through AKT, leading to nuclear factor-κB (NF-κB) activation, and cell invasion and metastasis. However, AKT is frequently activated in cancer. Thus, a key question is whether ASIC1-mediated cell signaling still takes place in the cancer cells carrying constitutively active AKT. In the present study, we show that among four prostate cancer cell lines tested, 22Rv1 cells express the highest level of phosphorylated AKT that is not impacted by acidosis. However, acidosis can still induce NF-κB activation during which extracellular signal-regulated kinase (ERK) serves as an alternative pathway for ASIC-mediated cell signaling. Inhibition of ERK by chemical inhibitors or small interfering RNAs suppresses the acidosis-induced NF-κB activity through regulation of the inhibitory subunit IκBα phosphorylation. Furthermore, suppression of ASIC1-mediated generation of reactive oxygen species (ROS) by ROS scavengers, such as glutathione or *N*-acetyl-cysteine causes a decrease in ERK phosphorylation and degradation of IκBα. Finally, ASIC1 is upregulated in a subset of prostate cancer cases and ASIC1 knockout by CRISPR/Cas9 significantly suppresses cell invasion, and castration resistance both *in vitro* and *in vivo*. Together, these results support the significance of ASIC1-ROS-ERK-IκBα-NF-κB axis in prostate tumorigenesis, especially in the constitutively active AKT background.

## Introduction

Hypoxic conditions, disorganized tumor vasculature, heterogeneous blood flow and increased glycolysis (Warburg effect) are a common feature for solid tumors, which often lead to an acidotic condition (acidosis).^1^ As low intracellular pH (pHi) is harmful, tumor cells have to employ various mechanisms to remove intracellular acids in order to maintain physiological pHi. These include V-type ATPases, Na^+^/H^+^ exchangers and those facilitated by carbonic anhydrases such as CA2, CA9 and CA12.^2^ As a result, the extracellular pH (pHe) becomes acidic. For example, pHe of solid tumors could reach 6.2–6.8.^3^ In some extreme cases, interstitial pH values could even be as low as 5.8.^4^ Although such acidic extracellular conditions are often harmful to normal cells, tumor cells seem to have adapted well and furthermore, tumor cells can even use acidosis as a signal to promote their invasion and metastasis.^1^ We recently reported that acid-sensing ion channels (ASICs) are expressed in a subset of breast cancer cases and breast cancer cell lines.^5^ More importantly, ASIC1 plays a critical role in breast cancer cell invasion and metastasis, which is in part through activation of AKT and nuclear factor-κB (NF-κB).

AKT is a key downstream target of phosphoinositide 3-kinase (PI3K)-mediated signaling pathway and it plays an important role in regulation of diverse cellular processes such as cell survival, cell cycle progression, metabolism and angiogenesis.^6^ Inappropriate activation of AKT has been reported in many types of human diseases, including cancer. Several known factors control AKT activity positively or negatively; a notable negative regulator is the tumor suppressor PTEN that is frequently mutated or deleted in cancer. In addition, reactive oxygen species (ROS) can deactivate several phosphatases, including PTEN, leading to activation of AKT.^7^ Activated AKT is capable of phosphorylating IκBα kinase (IKK), which is a kinase for IκBα, a direct inhibitor of NF-κB. Thus, AKT can serve as an upstream molecule for NF-κB.

NF-κB is a ubiquitously expressed pleiotropic transcription factor that can be activated in response to a number of stimuli, including acidosis.^8, 9, 10^ Under normal conditions, NF-κB stays in the cytoplasm as a heterotrimeric complex consisting of the subunits p50, p65 and the inhibitory subunit IκBα. In response to inducing stimuli, IκBα undergoes phosphorylation, ubiquitination and proteolytic degradation. The p65 subunit then undergoes phosphorylation and moves into the nucleus, where it binds to specific DNA sequence and can activate the transcription of hundreds of genes.^11^ In addition to AKT, IKK can also catalyze phosphorylation of IκBα. Aberrant regulation of NF-κB and the signaling pathways that control its activity often leads to inflammation, drug/radiation resistance and tumorigenic potential of cancer cells.^12^ As an AKT effector, NF-κB is critical to the acidosis-induced tumor initiation, progression and invasion.^5^ However, a question remains as to whether acidosis can still induce the cell signaling and impact different aspects of tumorigenesis in the highly active AKT background.

In the present study, we show that ERK, one of mitogen-activated protein kinases (MAPKs), serves as an alternative pathway through which ASIC1 and NF-κB are connected, especially in cancer cells carrying constitutively active AKT, leading to tumor cell growth and invasion.

## Results

### Acidosis induces NF-κB activity independent of AKT status

**Figure 1:**
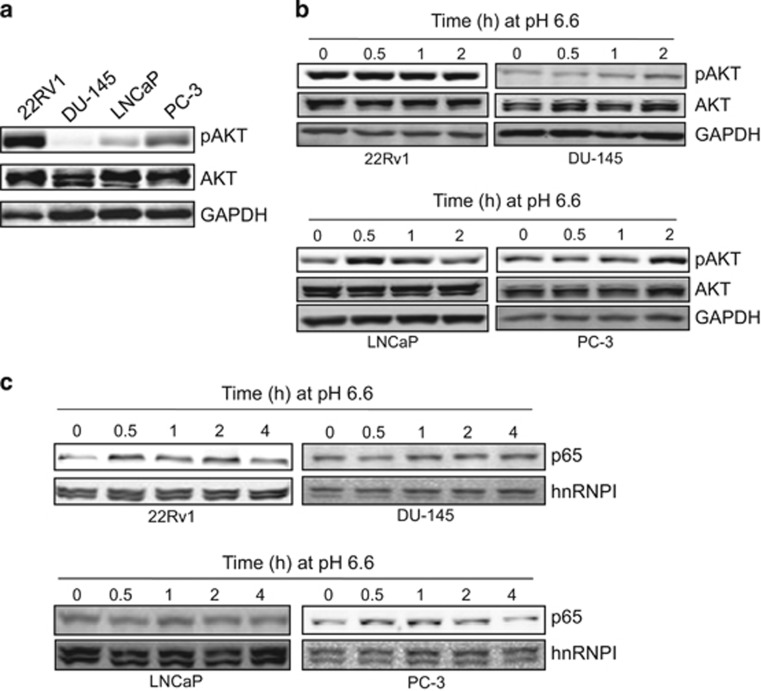
Acidosis induces nuclear translocation of NF-κB. (**a**), Expression of pAKT in 22Rv1, DU-145, LNCaP and PC-3 cells, as detected by western blot. (**b**) Detection of acidosis-induced AKT in selected prostate cancer cells. Note that no AKT activation is seen in 22Rv1 cells. (**c**) Nuclear localization of NF-κB in prostate cancer cells in response to acidosis. ‘Time 0 h' indicates that the cells were cultured at pH 7.4.

We recently showed that acidosis induces activation of AKT and NF-κB.^13^ However, AKT is often highly activated in cancer due to various mechanisms, including PTEN mutation or deletion. Hence, we asked whether acidosis-induced cell signaling still takes place. To this end, we first examined the pAKT level in prostate cancer cell lines 22Rv1, LNCaP, DU-145 and PC-3. It is evident that the pAKT level is highest in 22Rv1 cells among them (Figure 1a). Next, we determined the effect of acidosis on AKT activity. As acidosis induces AKT activation as early as 5 min after exposure to acidosis (pH 6.6) and the pAKT level reached a peak at 2 h,^13^ we treated cells at pH 6.6 for up to 2 h. Although we detected an increase in pAKT in LNCaP, DU-145 and PC-3, no AKT activation was seen in 22Rv1 cells (Figure 1b). For instance, in LNCaP cells AKT was induced at as early as 30 min; in PC-3 cells this AKT induction was seen at 2 h after exposure to acidosis. These results suggest that induction of AKT by acidosis is dynamic in different cell lines and different cell signaling pathways may be involved in this process. Next, we determined whether NF-κB is activated in response to acidosis because NF-κB is a key factor responsible for cell growth, invasion and metastasis. As shown in Figure 1c, not all cell lines responded to acidosis in the same way. For instance, in DU-145 cells NF-κB activation was seen at 1 h, whereas in PC-3 we detected NF-κB activation at 30 min. NF-κB activation was not obvious in LNCaP cells. Of interest was 22Rv1 with activation of NF-κB at as early as 30 min despite of a high level of base line AKT activity, suggesting that acidosis can still impact tumorigenesis in the cancer cells with constitutively active AKT.

### ASIC1 is required for acidosis-induced cell signaling

**Figure 2:**
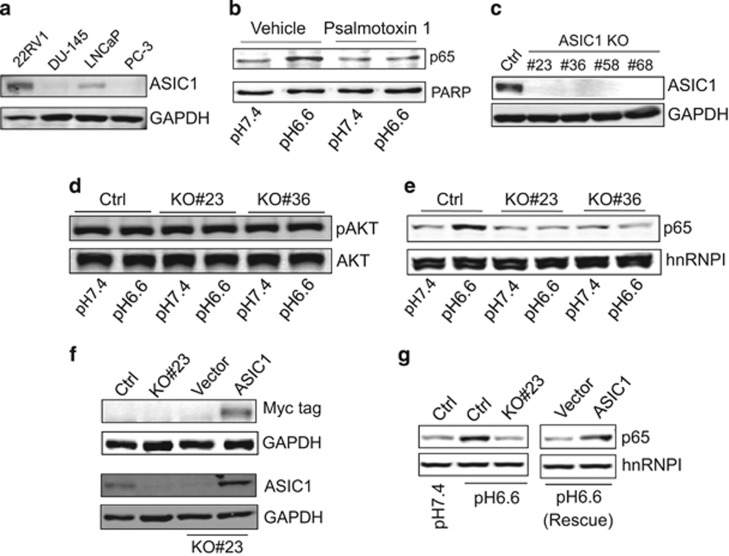
ASIC1 is required for acidosis-induced nuclear translocation of NF-κB. (**a**) Detection of ASIC1 expression in prostate cancer cell lines by western blot. (**b**) Suppression of NF-κB activity by ASIC1 inhibitor psalmotoxin 1 in response to acidosis. (**c**) Knockout (KO) of ASIC1. (**d**) ASIC1 KO has no effect on pAKT. Cells were treated at pH 6.6 for 2 h. (**e**) ASIC1 KO suppresses nuclear translocation of NF-κB. Cells were treated at pH 6.6 for 2 h. (**f**) Detection of ASIC1 (ASIC1-Myc-tag) in KO cells after re-expression. (**g**) Re-expression of ASIC1 restores the ability to activate NF-κB in response to acidosis. Cells were treated at pH 6.6 for 2 h.

As ASIC1 has been shown to be required for acidosis-induced cell signaling in breast cancer,^5^ we determined expressions of ASIC1 in these four prostate cancer cell lines. As shown in Figure 2a, two of these cell lines (22Rv1 and LNCaP) expressed a significant level of ASIC1; moreover, the ASIC1 level was much higher in 22Rv1 cells than in LNCaP cells. A similar trend was also seen at the messenger RNA level (Supplementary Figure S1). To determine the role of ASIC1 in acidosis-induced cell signaling, we treated the cells with specific ASIC1 inhibitor psalmotoxin 1 that was able to block acidosis-induced NF-κB activation in 22Rv1 cells (Figure 2b). Hence, we chose 22Rv1 cells to characterize the ASIC1-mediated cell signaling. First, we knocked out ASIC1 by CRISPR/Cas9 technology and dual guide RNA (gRNA) approach targeting exon 2 and 3 of ASIC1^5^ and obtained several knockout (KO) clones (Figure 2c). We chose two clones for further characterization. Although ASIC1 KO had no effect on the pAKT level in response to acidosis (Figure 2d), ASIC1 was critical to the acidosis-induced NF-κB activation (Figure 2e). There was little induction of NF-κB in either KO clone as compared with gRNA control. To further determine the role of ASIC1 in acidosis-induced NF-κB activation, we performed a rescue experiment, that is, re-expression of Myc-tagged ASIC1 in KO cells (Figure 2f). As expected, NF-κB activation was restored by ASIC1 (Figure 2g), demonstrating a critical role for ASIC1 in acidosis-mediated cell signaling. These results suggest that there may exist an alternative pathway bypassing AKT for acidosis-induced NF-κB activation.

### ERK is involved in ASIC1-mediated cell signaling

**Figure 3:**
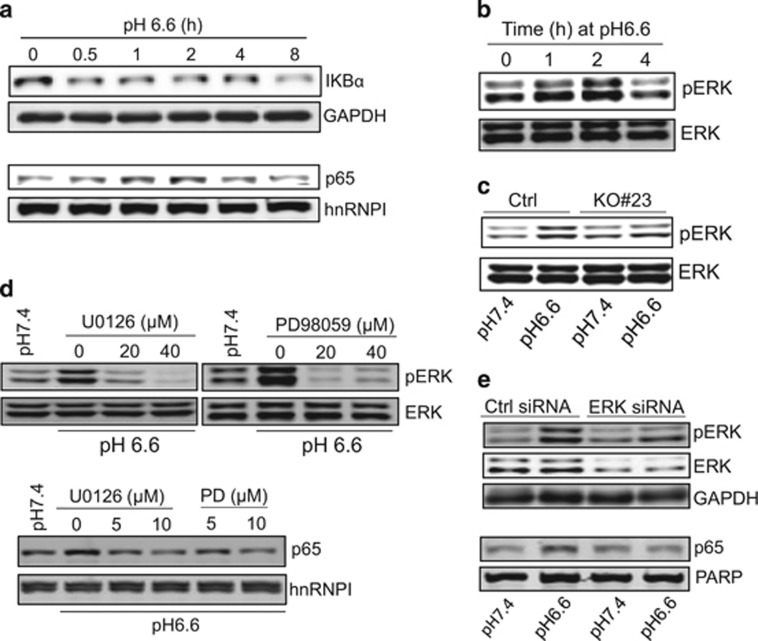
ERK is required for the acidosis-induced nuclear localization of NF-κB in 22Rv1 cells. For acidosis treatment, cells were cultured at pH 6.6 for 2 h before the cells were harvested. (**a**) Acidosis-induced nuclear localization of NF-κB is associated with a decrease in the IκBα level. (**b**) Acidosis induces pERK. (**c**) ASIC1 KO suppresses acidosis-induced ERK activation. (**d**) Suppression of ERK activity by ERK inhibitors U0126 and PD98059. Cells were treated with inhibitors at indicated concentrations for 1 h before acidosis. (**e**) Suppression of ERK activity by ERK siRNAs reduces nuclear localization of NF-κB.

IκBα serves as a NF-κB inhibitor and degradation of IκBα leads to nuclear translocation of NF-κB. Consistent with NF-κB activation, we detected the degradation of IκBα at as early as 30 min after acidosis (Figure 3a). To search for further upstream signaling molecules, we tested ERK that, like AKT, is also involved in a variety of important cellular processes such as proliferation, differentiation, motility, stress response, apoptosis and survival in response to stimuli such as mitogens, cytokines, growth factors and environmental stress. Once activation, ERK can regulate targets in the cytosol or translocate to the nucleus to regulate gene expression.^14^ As expected, we detected induction of ERK in response to acidosis in a time-dependent manner (Figure 3b). Of interest, ASIC1 is required for this acidosis-induced ERK activation. For instance, ASIC1 KO suppressed this induction (Figure 3c). To further determine the role of ERK in acidosis-induced NF-κB activation, we suppressed ERK by ERK inhibitors U0126 and PD98059. Both inhibitors blocked acidosis-induced NF-κB activation (Figure 3d; Supplementary Figure S2). The inhibition of NF-κB activity was seen at 5 μm for both inhibitors. Similarly, ERK small interfering RNAs (siRNAs) also suppressed NF-κB activation (Figure 3e). These results suggest a critical role for ERK in ASIC1-mediated cell signaling in response to acidosis.

### ERK is required for IκBα phosphorylation through ROS

**Figure 4:**
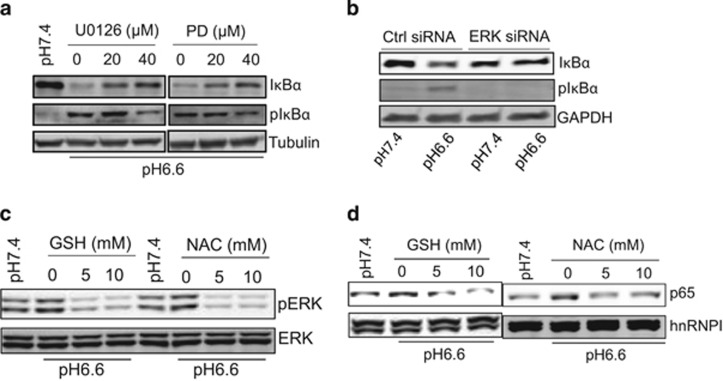
Acidosis induces phosphorylation of IκBα through ERK in 22Rv1 cells. For acidosis treatment, cells were cultured at pH 6.6 for 2 h before the cells were collected. (**a**) Suppression of ERK by ERK inhibitors reduces acidosis-induced IκBα degradation and IκBα phosphorylation. Cells were treated with U0126 and PD98059 at indicated concentrations for 1 h before acidosis. (**b**) Suppression of ERK activity by ERK siRNAs reduces acidosis-induced IκBα degradation and IκBα phosphorylation. (**c**) Both GSH and NAC suppress ERK activity. (**d**) Both GSH and NAC suppress nuclear localization of NF-κB.

Moreover, this acidosis-induced IκBα degradation was also blocked by ERK inhibitors (Figure 4a). Like other types of stress, acidosis induces phosphorylation of IκBα^15^ that leads to IκBα degradation.^16^ In consistent with this finding, ERK inhibitors suppressed phosphorylation of IκBα (Figure 4a). Similarly, ERK siRNAs also blocked the acidosis-induced phosphorylation of IκBα such that the level of IκBα was restored (Figure 4b). As acidosis can cause generation of ROS,^17, 18^ we tested the role of ROS in acidosis-induced activation of ERK. We treated the cells with two ROS scavengers glutathione (GSH) and *N*-acetyl-cysteine (NAC). As shown in Figure 4c, both GSH and NAC reduced the level of pERK induced by acidosis. Moreover, both GSH and NAC reduced the nuclear p65 level (Figure 4d).

### Acidosis increases invasion ability of 22Rv1 cells through ASIC1

**Figure 5:**
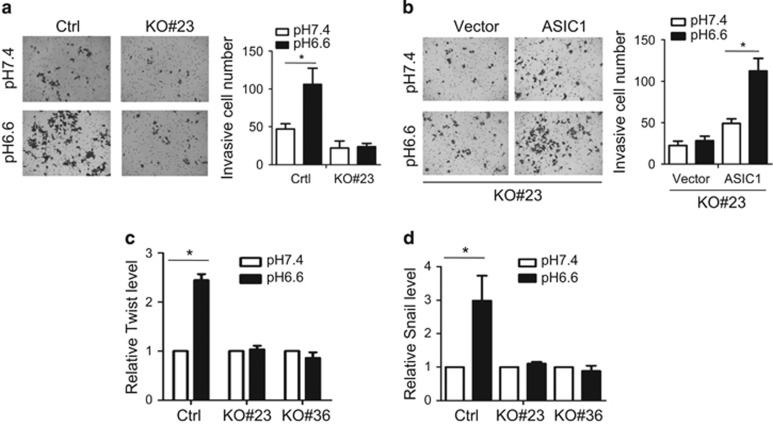
ASIC1 promotes acidosis-induced cell invasion and expression of Twist and Snail. (**a**) ASIC1 KO suppresses acidosis-induced cell invasion. Invasion assay was detailed in Materials and methods. Invasive cell number is the average of the number from five fields. (**b**) Re-expression of ASIC1 in KO cells restores the invasion ability. (**c**, **d**) ASIC1 KO suppresses acidosis-induced Twist and Snail, respectively. Cells were cultured at pH 6.6 for 2 h before the cells were collected for RNA isolation and quantitative PCR with reverse transcription. Values are mean±s.e. (*n*=3), *P<0.05.

Given the importance to ERK and NF-κB in cancer, we tested the effect of ASIC1 KO on cell invasion by transwell invasion assays. There was an increase in cell invasion by acidosis, and ASIC1 KO significantly reduced acidosis-induced invasive cell number as compared with gRNA control (Figure 5a, left). For example, for gRNA control, average invasive cell number was <50 for pH 7.4; this number was increased to over 100 for pH 6.6. In the ASIC1 KO group, the invasive cell number was under 20 for both pH 6.6 and pH 7.4 (Figure 5a, right). Rescue experiments revealed that ASIC1 was able to restore the ability to increase invasiveness (Figure 5b). In consistent with these findings, we found that as expected, acidosis increased expression of Twist and Snail (Figures 5c and d), both of which have been implicated in cell invasion and metastasis.^19^ In contrast, there was no increase of Twist and Snail in ASIC1 KO cells in response to acidosis.

### ASIC1 promotes tumor cell growth and castration resistance

**Figure 6:**
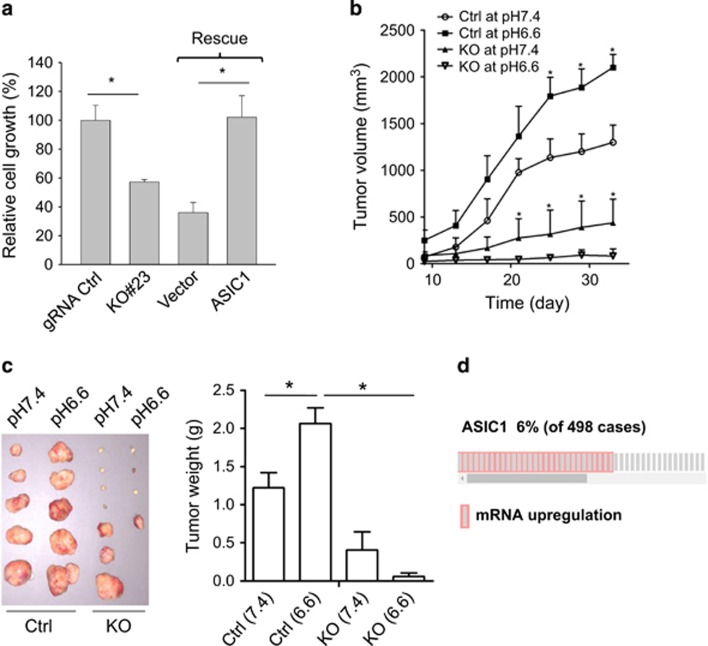
ASIC1 is required for cell growth in androgen-free medium and tumor growth in castrated male mice. (**a**), ASIC1 KO (#23) suppresses cell growth; re-expression of ASIC1 increases cell growth in androgen-free medium. Values are mean±s.e. (*n*=3), *P<0.05. (**b**) ASIC1 KO (#23) causes tumor growth inhibition. Two tumors for pH 6.6 gRNA control group were collected at day 19 and another two tumors for pH 7.4 gRNA control group were collected at day 23 because they reached 2 cm in diameter; (**c**) ASIC1 KO (#23) reduces tumor load. Two tumors for pH 6.6 gRNA control group and two for pH 7.4 gRNA control group harvested early were not pictured here (left), but they were included in the calculation of average tumor weight (right). The rest tumors were harvested at day 33 after tumor cell injection. Values are mean±s.e. (*n*=7), *P<0.05. (**d**) Analysis of prostate adenocarcinoma data set (The Cancer Genome Atlas, Provisional) at cBioPortal (http://www.cbioportal.org/) indicates 6% of cases with upregulation of ASIC1. Query Language (OQL) setting was ‘ASIC1: EXP>1.5'.

As ASIC1 is at top of this ASIC1-ROS-ERK-IκBα-NF-κB axis, we determined the consequence of ASIC1 KO. Cell growth assays indicated that ASIC1 slightly suppressed cell growth in regular medium (Supplementary Figure S3). However, in androgen-free medium ASIC1 KO significantly suppress cell growth, implicating ASIC1 in castration resistance (Figure 6a; Supplementary Figure S3). Therefore, we focused on the effect of ASIC1 on castration resistance. Rescue experiments further confirmed that re-expression of ASIC1 restored cell growth in androgen-free medium (Figure 6a). To further determine the role of ASIC1 in castration resistance, we performed xenograft animal tumor models using castrated severe combined immunodeficiency male mice. As shown in Figures 6b and c, ASIC1 KO suppressed tumor growth and tumor weight. Finally, by interrogating The Cancer Genome Atlas prostate adenocarcinoma data set at cBioPortal (http://www.cbioportal.org/),^20, 21^ we found that ASIC1 is upregulated in a subset of patient populations (6% among 498 cases; Figure 6e). Furthermore, when all four ASIC genes (ASIC1–4) were combined, they were upregulated in 18% of 498 cases (Supplementary Figure S4). Together, these results suggest that ASIC1 plays a role in prostate tumorigenesis and castration resistance.

## Discussion

AKT and ERK are two well-known signaling molecules downstream of receptor tyrosine kinase, and play a critical role in cell growth, proliferation and survival in response to growth factors or stress. Although AKT and ERK pathways can cross talk each other, they are relatively independent. For instance, AKT signaling involves PI3K, whereas ERK signaling is through RAS/RAF. However, they converge on survival signals. Thus, it is important to determine how they respond to environmental stimuli in different cell cellular content.

Through a long period of co-evolution with the host, tumor cells have adapted to acidic microenvironment and as such they become more aggressive.^22, 23, 24^ Early studies have implicated ASICs in the growth and migration of glioma cells.^25, 26^ For instance, acidosis causes activation of AKT and NF-κB, and generation of ROS;^8, 10, 13^ expression of ASIC1 and ASIC2 could induce glioma cation current, whereas suppression of this conductance decreases glioma growth and cell migration.^25^ Our recent study indicates that ASIC1 is critical to breast cancer invasion and metastasis through the ROS-AKT-NF-κB pathway.^5^ However, little is known whether there is any alternative acidosis-induced pathway, leading to NF-κB activation and cell invasion. The present study provides evidence that ASIC1 plays a critical role in response to acidosis in prostate cancer. In particular, our study suggests that ERK serves as a key player in ASIC1-mediated cell signaling in the highly active AKT background.

It is well known that tumor cells often carry highly active AKT activity that can be attributed to many factors, including deletion or mutation of PTEN^27^ or mutations in the PI3K genes.^28^ In this scenario, acidosis-induced cell signaling can take alternative pathways such as MAPK pathway. Like AKT, MAPKs are a highly conserved family of serine/threonine protein kinases involved in a variety of important cellular processes, such as proliferation, differentiation, motility, stress response, apoptosis, and survival. Extracellular stimuli, such as mitogens, cytokines, growth factors and environmental stress, cause a cascade of MAPK signaling through phosphorylation of MAP3K, MAP2K (for example, mitogen-activated protein kinase kinase (MEK)) and MAPK (for example, ERK). ERK serves as a substrate for MEK. Once activation by MEK, ERK can regulate a variety of downstream targets. Several lines of evidence from this study suggest that ERK plays a critical role in acidosis-induced NF-κB activation in cancer cells carrying a highly active AKT. (1) Acidosis activates ERK in a time-dependent manner; (2) ERK inhibitors or ERK siRNAs suppress the acidosis-induced NF-κB activation; and (3) ERK inhibitors or ERK siRNAs suppress the acidosis-induced IκBα phosphorylation. These findings are in line with the report that ASIC1 is also required for ERK activation in glioma cells.^29^

Our previous study suggests the involvement of acidosis-induced ROS production in breast cancer cells, leading to activation of NF-κB.^13^ As the present study reveals that both GSH and NAC suppress the acidosis-induced ERK activation, and subsequently inhibit nuclear localization of NF-κB, it may suggest that ROS acts at the upstream of ERK. Although the precise underlying mechanism still remains to be determined yet, it is possible that ROS may activate MAPK pathways through oxidative modifications of MAPK signaling proteins and inactivation and/or degradation of MAP kinase phosphatases.^30^ In support of this notion, ROS can alter protein structure and function by modifying critical amino-acid residues of proteins.^31^ Similarly, PTEN is a phosphatase and it can undergo inactivation (oxidation) by ROS.^32^ We have shown that GSH and NAC can block ROS-induced PTEN inactivation in response to acidosis.^13^ In addition, oxidant H_2_O_2_ may activate MAPK pathways via activation of growth factor receptors in several cell types.^33^

Although AKT and ERK are distinct pathways, they also cross talk each other. In particular, they not only negatively regulate each other's activity, but they cross activate each other.^34^ More importantly, they can share same substrates. For instance, both AKT and ERK can activate ER by phosphorylation in breast cancer.^35, 36^ In support of this notion is our finding that ASIC1-mediated NF-κB activation can bypass AKT through ERK, especially in the case that AKT is constitutively activated. They converge on NF-κB activation regardless of AKT status.

The clinical significance of ASIC1 is supported by the findings that ASIC1 is upregulated in a subset of prostate cancer cases. Furthermore, ASIC1 also contributes to castration resistance. For example, although ASIC1 KO suppresses cell growth in normal medium, this suppression is more significant under androgen deprivation. However, the underlying mechanism still remains to be determined. Accumulating evidence indicates that multiple factors can contribute to castration resistance, including androgen receptor, c-Myc overexpression, upregulation of PI3K/AKT and RAS/MAPK.^37^ ERK is frequently activated in prostate cancer samples, especially metastatic samples.^38^ Overexpression of nuclear receptor coactivator 2 along with loss of PTEN can result in activation of ERK, promoting tumor malignance in murine prostate tumor model.^39^ Thus, it is conceivable that ERK activation may contribute to castration resistance especially when tumor microenvironment becomes acidic.

**Figure 7:**
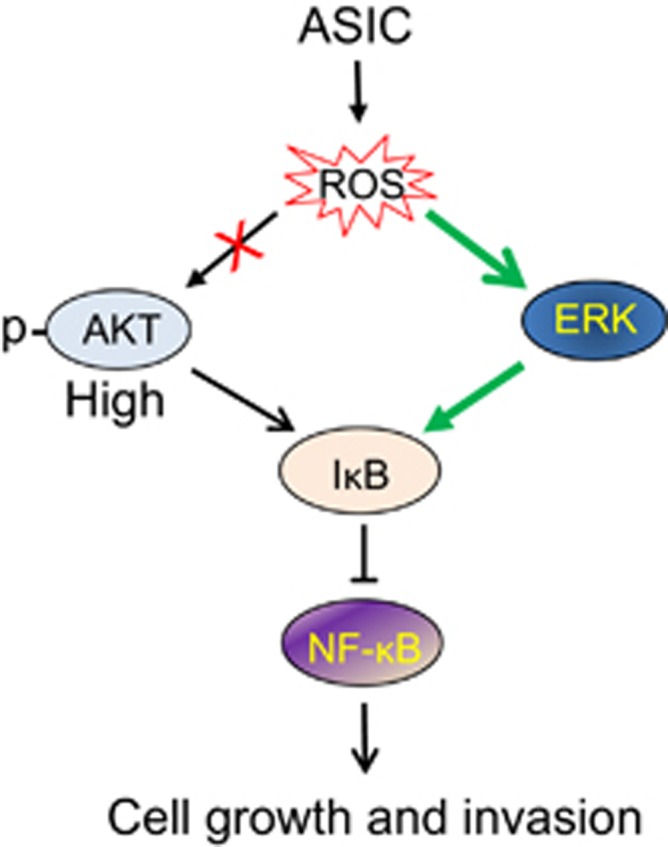
A working model for acidosis-induced NF-κB activation through ERK in the highly active AKT background. See text for explanation.

In summary, our results suggest that ERK serves as an alternative pathway in ASIC1-mediated cell signaling in response to acidosis under constitutively active AKT background (Figure 7). ASIC1 sits at the top of this cascade. Acidic tumor microenvironment can induce ROS. Although IKK is a well-known factor that can phosphorylate IκBα, other factors such as AKT can also regulate IκBα activity. The present study suggests that in the presence of highly active AKT, tumor cells can bypass AKT and acidosis-induced ROS may activate ERK (phosphorylation) possibly through deactivation of certain phosphatases. Subsequently, the activated ERK may facilitate IκBα phosphorylation directly or indirectly, leading to nuclear localization of NF-κB. As a cancer promoter, NF-κB can activate a larger number of genes^40^ important to cell growth, migration, invasion and metastasis. Therefore, this ASIC1-ROS-ERK-IκBα-NF-κB axis may provide an opportunity for intervention in cancer therapy.

## Materials and methods

### Regents

Sources of primary antibodies were: p65 (D14E12), p-IĸBα (5A5), IĸBα (L35A5), pERK (D13.14.4E), ERK (137F5), pAKT (D9E), AKT (C67E7), p-IKKα (16A6) and PARP (46D11) from Cell Signaling (Danvers, MA, USA); ASIC1 (clone N271/44) from UC Davis (Davis, CA, USA), sold by Antibodies Inc (Davis, CA, USA); Myc-tag from Applied Biological Materials (ABM; Richmond, BC, Canada); α-tubulin, GAPDH and ERK (66192-1-Ig) from Proteintech (Rosemont, IL, USA); and hnRNP I (SH54) from Santa Cruz Biotechnology (Dallas, TX, USA). Secondary antibodies conjugated with IRDye 800CW or IRDye 680 were purchased from LI-COR Biosciences (Lincoln, NE, USA). PCR primers were purchased from IDT (Coralville, IA, USA). ERK siRNAs, U0126 and PD98059 were purchased from Cell Signaling. GSH and NAC were obtained from Sigma-Aldrich (St. Louis, MO, USA). Psalmotoxin 1 was purchased from Peptides International (Louisville, KY).

### Cell culture

Prostate cancer cell lines 22RV1, DU-145, LNCaP and PC-3, and HEK293T were obtained from American Type Culture Collection (Manassas, VA, USA) and were authenticated by DDC Medical (http://www.ddcmedical.com) using the short tandem repeat profiling method. 22RV1, DU-145, LNCaP and PC-3 cells were cultured in RPMI-1640 medium with 10% fetal bovine serum (FBS) and 2 mM glutamine. HEK293T cells were cultured in DMEM with 10% FBS. For androgen-deprivation cells were grown in phenol free RPMI-1640 supplemented with charcoal stripped 5% FBS (Sigma-Aldrich). All media contained 2 mM glutamine, 100 units of penicillin per ml and 100 mg of streptomycin per ml. The pH of the acidic medium was adjusted to 6.6 with 4-(2-hydroxyethyl)-1-piperazineethanesulfonic acid (HEPES) and 1,4-piperazinediethanesulfonic acid (PIPES).

### Plasmid construction

KO constructs using the dual gRNA approach^41^ targeting ASIC1 exon 2 and 3, and donor for ASIC1 were described previously.^5^ ASIC1 expression vector used for rescue experiments was constructed by cloning ASIC1-coding region into pCDH-Myc by Cold Fusion kit (System Biosciences, Mountain View, CA, USA). All PCR products were verified by DNA sequencing.

### Transfection

Transfection of siRNAs was carried out using RNAfectin from ABM (Richmond, BC, Canada) according to the manufacturer's instructions. In brief, cells were seeded at 20–30% density in 12-well plates the day before transfection. Next day, control siRNAs or target gene siRNAs were mixed with RNAfectin and then added to cell culture at 100 nM. Sixteen hours later, the transfection medium was replaced with fresh medium.

### Invasion assay

Invasion assay was used for measuring the invasion ability of 22Rv1 cells in BioCoat invasion system from BD Biosciences (San Jose, CA). The cells were cultured in either acidic or normal medium for 2 h. Then, cells (2 × 10^4^) in 500 μl serum-free medium were added to the up chambers and the low chambers contained medium with 10% FBS. After 24 h, cells in the upper membrane were wiped away, and the cells in the lower membrane were immersed in 95% ethanol followed by stain with crystal violet. Cells were counted in five fields.

### Cell growth assay

22Rv1 cells (2 × 10^4^) were seeded in 12-well plates under either normal medium or androgen-free medium. The cells were collected, trypan blue stained and counted at day 4 as described.^42^ The relative cell growth was calculated by fold changes of cell numbers.

### Real time quantitative PCR with reverse transcription

Total RNA was isolated using Direct-zol RNA MiniPrep (Zymo Research, Irvine, CA, USA) per the manufacturer's protocol and 0.5 μg RNA was used to synthesize complementary DNA by RevertAid Reverse Transcriptase (Fisher Scientific, Pittsburgh, PA, USA) with random primer mix (New England BioLabs, Ipswich, MA, USA) in 20 μl reaction. The resultant complementary DNA was used for quantitative PCR reactions. To specifically detect expression of Twist and Snail, we used the SYBR Green method with primers described in Supplementary Table 1. β-actin was used as an internal control. Delta-delta Ct values were used to determine their relative expression as fold changes, as previously described.^41^

### Western blot analysis

Cytoplasmic, nuclear or whole cellular extract were prepared to detect levels of proteins, and western blot analysis was carried out as described earlier.^13^

### Animal work

The animal studies were conducted in accordance with National Institutes of Health animal use guidelines and the experimental protocol approved by the UMMC's Animal Care and Use Committee. Male severe combined immunodeficiency mice at 5–6 week old purchased from Charles River (Wilmington, MA, USA) were first castrated, and 1 week later tumor cells were injected subcutaneously into these mice with 1 million cells containing 50% matrigel per spot. Four groups of mice consisted of ASIC1 KO or vector control cells grown at pH 7.4 and pH 6.6, respectively, for 2 h before they were collected. Tumor growth was monitored every other 2 days and collected at day 33 after injection. Tumor volumes were calculated as: (*π*/6) × (length × width^2^).

### Statistical analysis

Comparisons between groups were analyzed using the Student's *t*-test (two groups) or a one-way analysis of variance followed by *post hoc* Tukey test (multiple groups). The Welch's correction of *t*-test was performed when the variances in two samples were unequal. Two-sided *P<*0.05 were considered as significant.
