# Supplementary material for: ERK-mediated NF-κB activation through ASIC1 in response to acidosis
Source: Oncogenesis. 2016 Dec 12;5(12):e279–. doi: 10.1038/oncsis.2016.81 (PMC5177778; doi:10.1038/oncsis.2016.81)

Supplementary Table 1. Primers in real time PCR

ASIC1	5’-GTG GCT GCC TCT CAG CTT AC-3’
	5’-CCA GCT CGG TCG TAG AAC TC-3’
Snail1	5’-TTT ACC TTC CAG CAG CCC TA-3’
	5’-CCT CAT CTG ACA GGG AGG TC-3’
Twist1	5’-GGA GTC CGC AGT CTT ACG AG-3’
	5’-TGG AGG ACC TGG TAG AGA AA-3'

Fig. S1

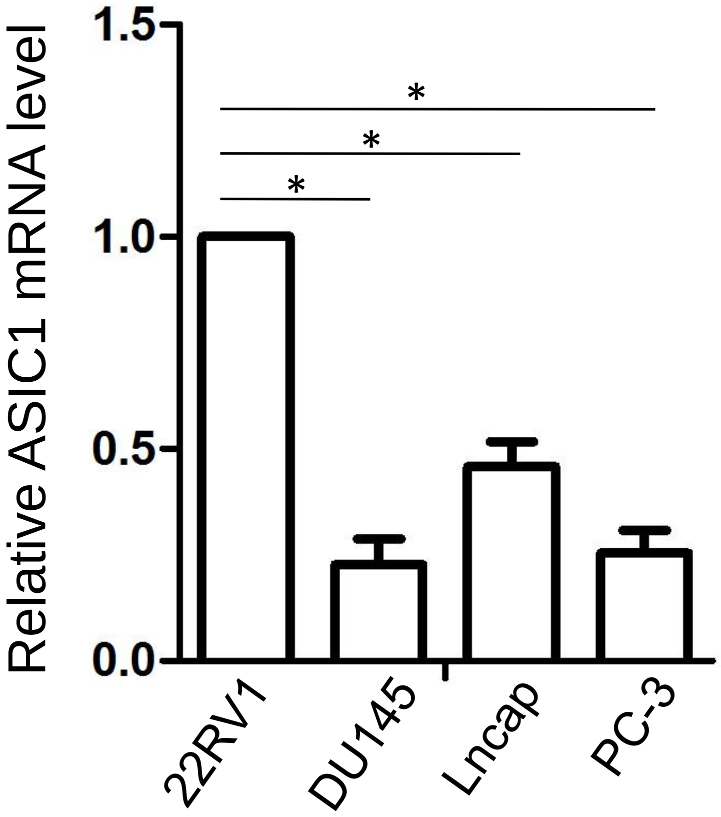

Fig. S1. Detection of ASIC1 mRNA by qRT-PCR in four prostate cancer cell lines. Values are mean ± SE (n = 3). *, $p < 0.05$.

Fig. S2

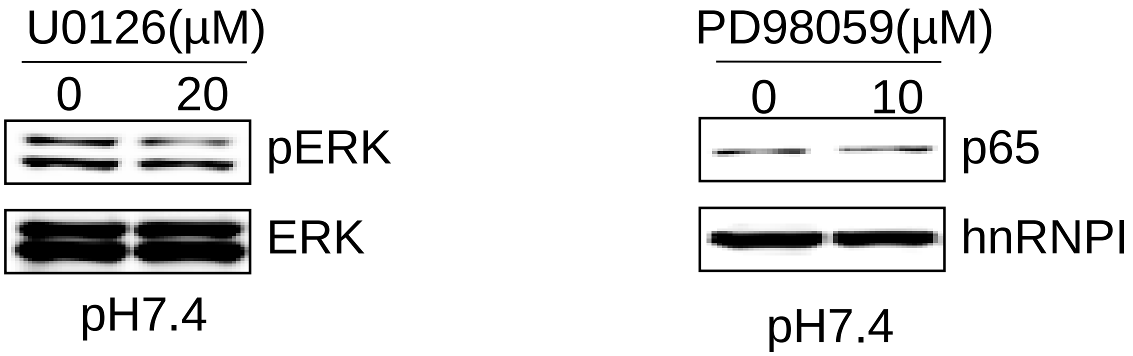

Fig. S2. ERK inhibitors U0126 and PD98059 have little effect on pERK or p65 at pH 7.4

Fig. S3

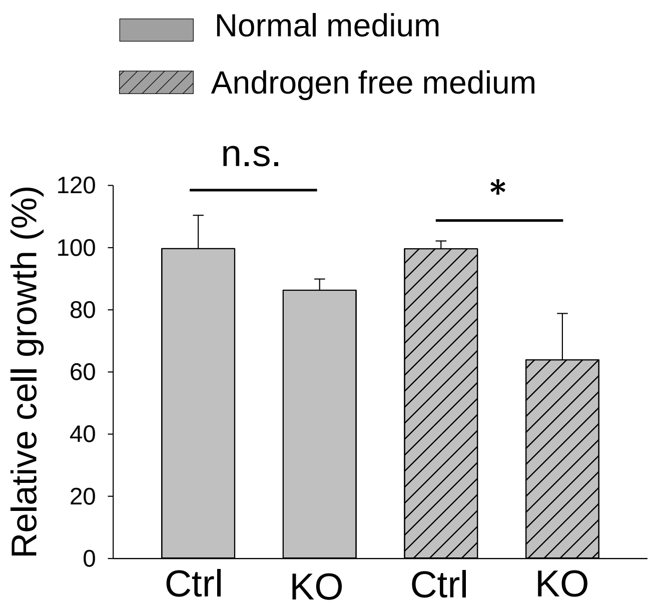

Fig. S3. ASIC1 is required for cell growth in androgen free medium. Cells (gRNA control and ASIC1 KO #23) were grown in either normal or androgen free medium. Cell number was counted at day 4. Values are mean ± SE (n = 3). *, $p < 0.05$.

Fig. S4

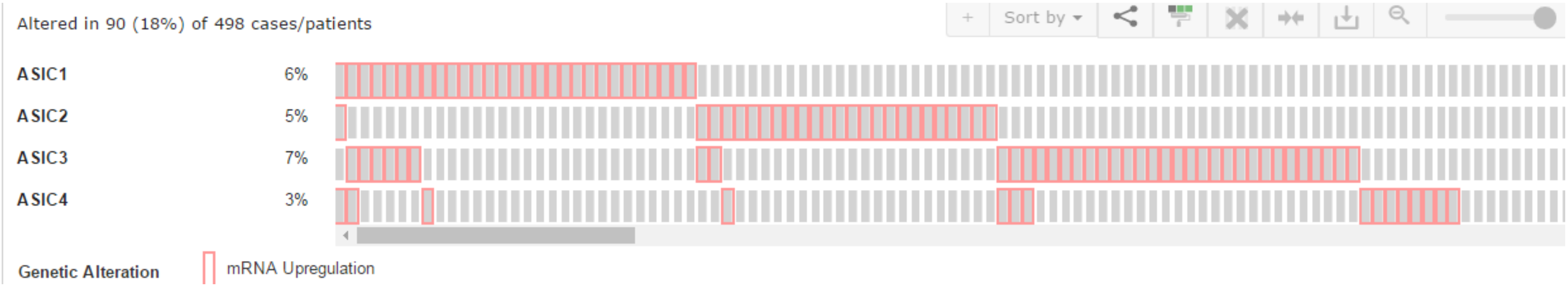

Fig. S4. Analysis of prostate adenocarcinoma dataset (TCGA, Provisional) at cBioPortal (http://www.cbioportal.org/) identified 18% of cases with upregulation of ASIC1~4 . Query Language (OQL) setting was “ EXP>1.5”

Additional supplemental information

Quantification of Western blots

Fig.1A

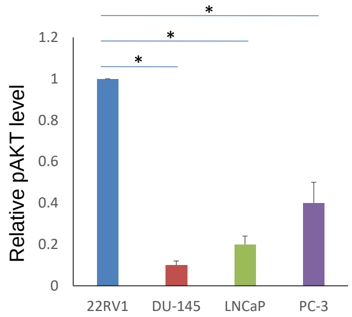

Fig.1B

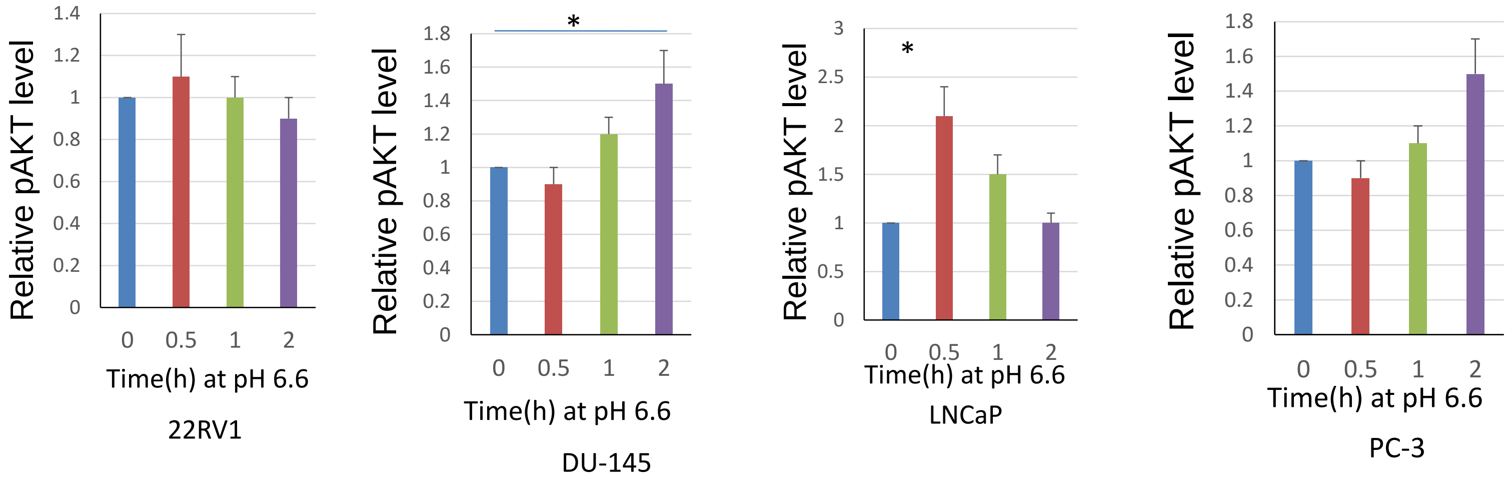

Fig.1C

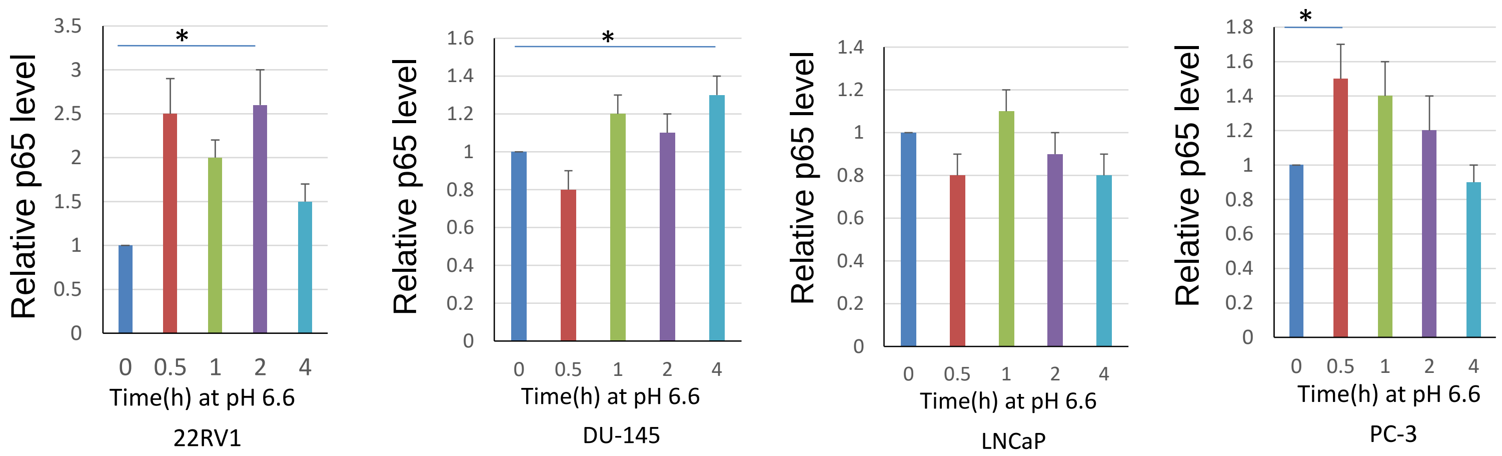

Fig.2A

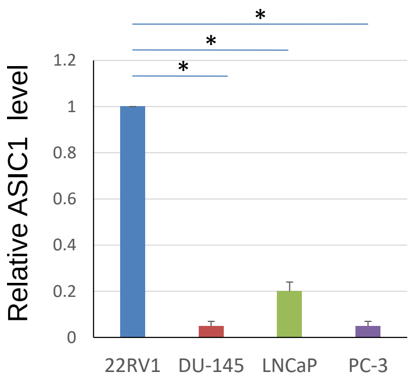

Fig.2B

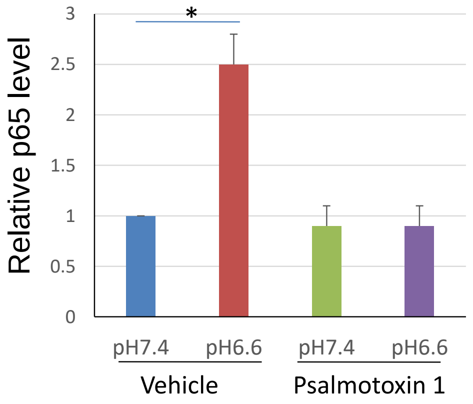

Fig.2D

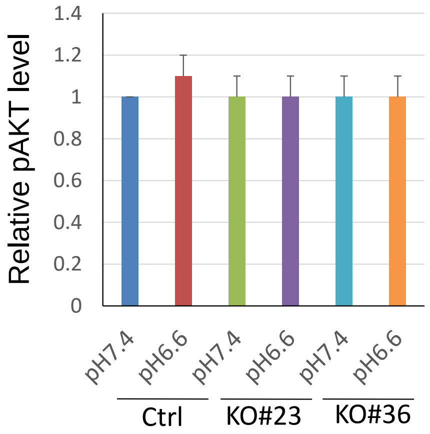

Fig.2E

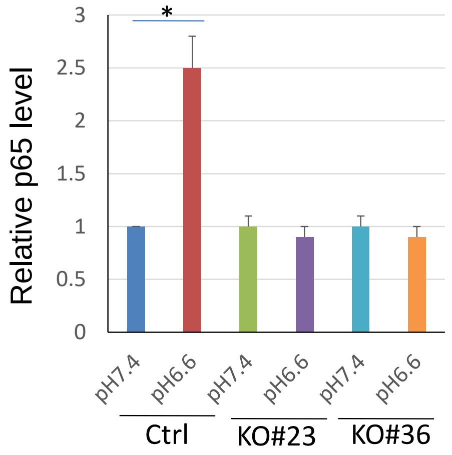

Fig.2G

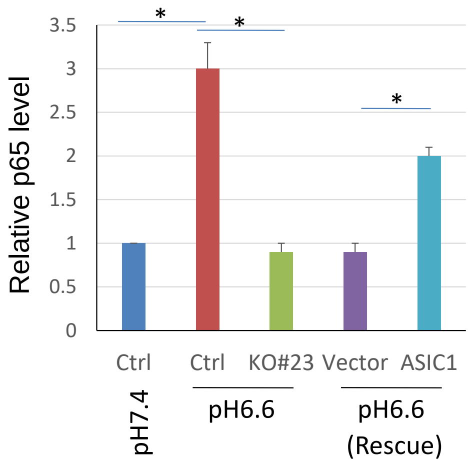

Fig.3A

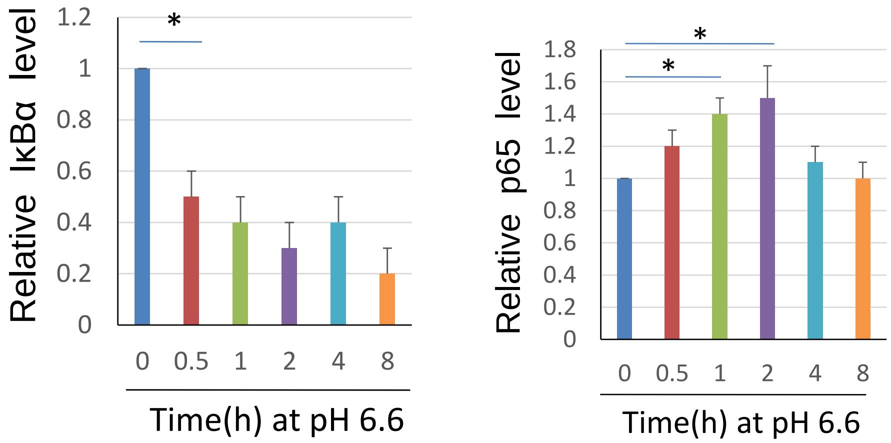

Fig.3B

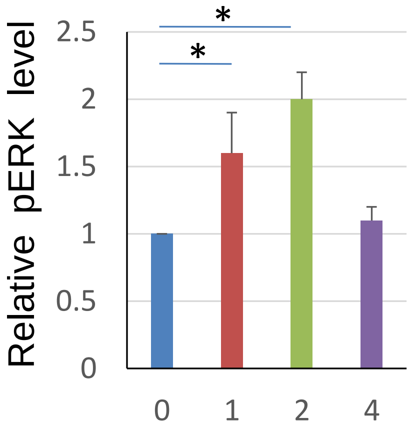

Fig.3C

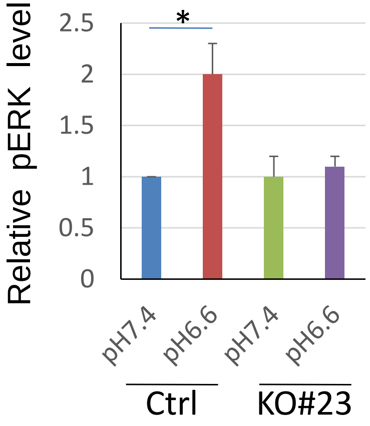

Fig.3D

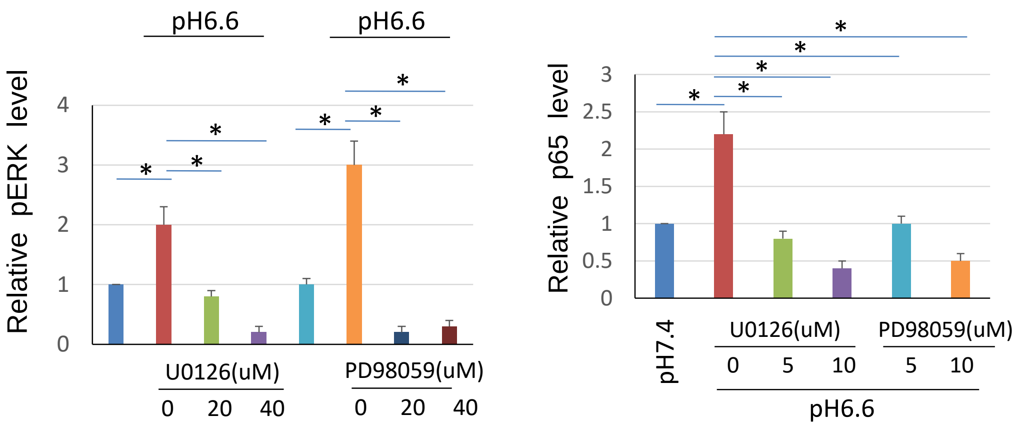

Fig.3E

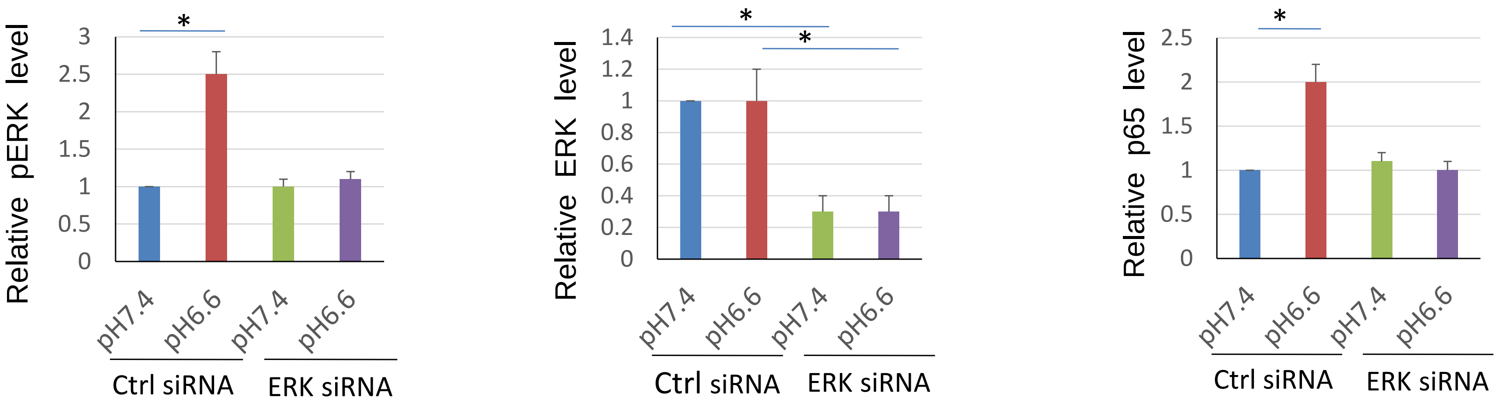

Fig.4A

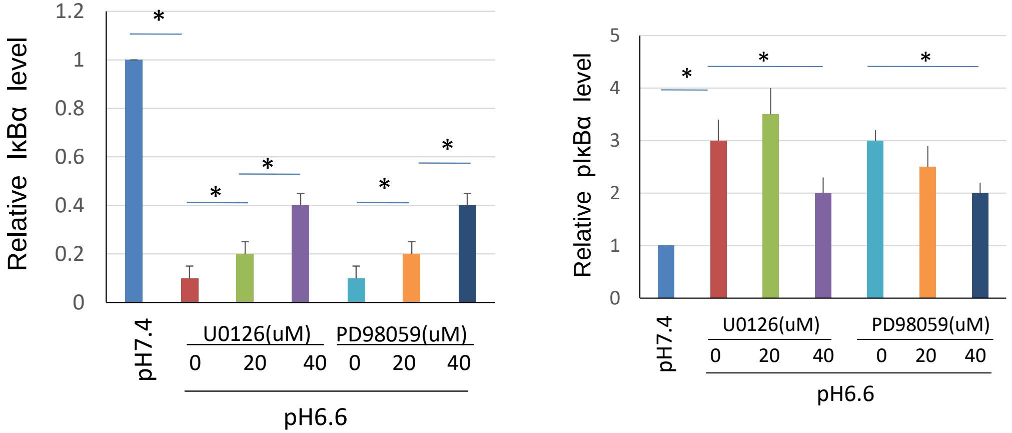

Fig.4B

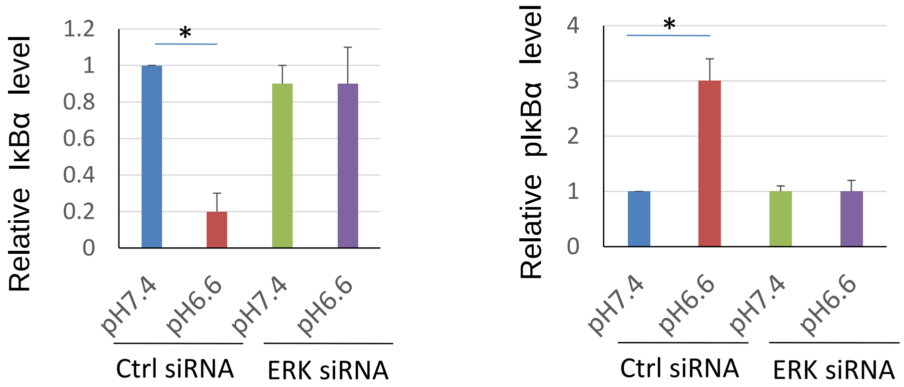

Fig.4C

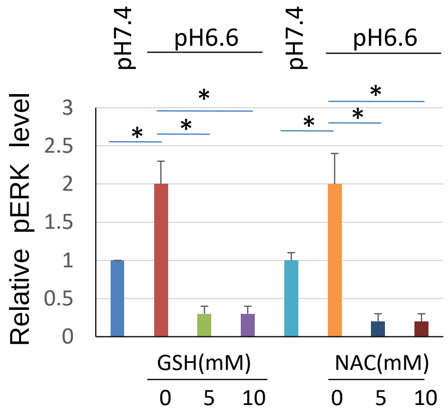

Fig.4D

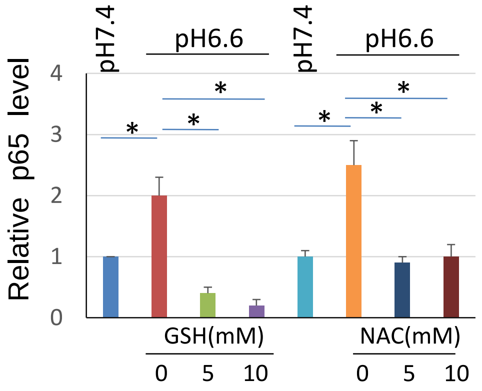

Supplement: Supplementary Information [file oncsis201681x1.pdf]
